# Osteoarthritis-Like Changes in Bardet–Biedl Syndrome Mutant Ciliopathy Mice (*Bbs1*^M*390*R/M*390*R^): Evidence for a Role of Primary Cilia in Cartilage Homeostasis and Regulation of Inflammation

**DOI:** 10.3389/fphys.2018.00708

**Published:** 2018-06-19

**Authors:** Isaac D. Sheffield, Mercedes A. McGee, Steven J. Glenn, Da Young Baek, Joshua M. Coleman, Bradley K. Dorius, Channing Williams, Brandon J. Rose, Anthony E. Sanchez, Michael A. Goodman, John M. Daines, Dennis L. Eggett, Val C. Sheffield, Arminda Suli, David L. Kooyman

**Affiliations:** ^1^Department of Physiology and Developmental Biology, Brigham Young University, Provo, UT, United States; ^2^Departments of Pediatrics and Ophthalmology, University of Iowa, Iowa City, IA, United States

**Keywords:** Bardet–Biedl syndrome, Bbs, osteoarthritis, primary cilia, inflammation

## Abstract

Osteoarthritis (OA) is a debilitating inflammation related disease characterized by joint pain and effusion, loss of mobility, and deformity that may result in functional joint failure and significant impact on quality of life. Once thought of as a simple “wear and tear” disease, it is now widely recognized that OA has a considerable metabolic component and is related to chronic inflammation. Defects associated with primary cilia have been shown to be cause OA-like changes in Bardet–Biedl mice. We examined the role of dysfunctional primary cilia in OA in mice through the regulation of the previously identified degradative and pro-inflammatory molecular pathways common to OA. We observed an increase in the presence of pro-inflammatory markers TGFβ-1 and HTRA1 as well as cartilage destructive protease MMP-13 but a decrease in DDR-2. We observed a morphological difference in cartilage thickness in Bbs1^*M390R/M390R*^ mice compared to wild type (WT). We did not observe any difference in OARSI or Mankin scores between WT and *Bbs1*^*M390R/M390R*^ mice. Primary cilia appear to be involved in the upregulation of biomarkers, including pro-inflammatory markers common to OA.

## Introduction

Osteoarthritis (OA) is a debilitating disease characterized by joint pain and effusion, loss of mobility, and deformity that may result in functional joint failure and significant impact on quality of life (1–3). It is also one of the most common chronic diseases in the United States, with recent estimates suggesting that more than 27 million adults suffer from clinical OA in this country. Vulnerable populations include the elderly, where 80% of people above 65 years of age are symptomatic for OA (Felson et al., 1988; Guccione, 1994; Lawrence et al., 2008), and the obese. As upward obesity trends continue it is probable that an increase in clinical OA will also be observed (1–3). There is currently no cure, and treatment options are minimal, with pain management and surgical joint replacement procedures being the only reprieve.

Once thought of as a passive “wear and tear” disease, it is now widely recognized that OA has a considerable metabolic component. It has been shown that whether the onset of the disease is due to aging, obesity, joint misalignment, acute injury, or genetic predisposition, all result in the activation of a common molecular pathway (Murwantoko et al., 2004; Lawrence et al., 2008; Polur et al., 2010; Holt et al., 2012; Larkin et al., 2013). Previous work has successfully identified key mediators in this pathway, including transforming growth factor beta 1 (TGF-β1), high temperature receptor A-1 (HTRA1), discoidin domain-containing receptor 2 (DDR-2), matrix metalloprotease 13 (MMP-13), and other matrix-degrading enzymes that are secreted by chondrocytes and contribute to the degradation of articular cartilage. Stress to the chondrocyte leads to an increase in TGF-β1-mediated chondroplasia but also has the potential to stimulate osteogenesis. Perhaps to offset this phenomena, HTRA1 is secreted to cleave TGF-β1. Being a non-specific serine protease, HTRA-1 also cleaves proteins of the pericellular matrix and brings the collagenous matrix in contact with the surface of chondrocytes, binding to DDR2 and subsequently increasing production of MMP-13, which specifically cleaves the type II collagen, among other proteins, making up the majority of articular cartilage.

Activation of *MMP-13* gene expression appears to occur through a number of molecular pathways that work through either inflammation or primary cilia. That is not to say there are not some common themes. Stress-inducible nuclear protein 1 (NUPR1) has been shown to regulate MMP-13 expression *in vitro* (Yammani and Loeser, 2014). Yammani and Loeser (2014) showed that NUPR1, expressed in cartilage, is required for expression of MMP-13 via IL-1β. This might be a pathway for the catabolic effects of OA to be mediated through inflammation. This is especially interesting in light of the study done by Xu et al. (2015) in which they analyzed differential expression of genes in cartilage involved in OA and rheumatoid arthritis (RA). While these researchers identified multiple genes associated with the regulation of MMPs, the predominant ones were associated with inflammation. This might give greater credence for the role of early inflammatory signals (i.e., AGEs, IL-1) in the initiation and progression of OA. While more obvious, a similar role for inflammation appears to be present in RA. Araki et al. (2016) reported that histone methylation and the binding of signal transducer activator of transcription 3 (STAT3) was associated with RA and OA. They report that histone H3 methylation is associated with elevated expression of MMP-1, -3, -9, and -13. However, STAT3 was shown to increase expression, either spontaneous or IL-6 activated, of MMPs 1, 3, and 13 but not 9. As previously indicated, primary cilia appear to also be involved in OA. Sugita et al. (2015) reported that transcription factor hairy and enhancer of split-1 (Hes1) is involved in the upregulation of expression of MMP-13. Normally Hes1 acts as a transcriptional repressor but under the influence of calcium/calmodulin-dependent protein kinase 2 (CaMK2) it becomes a transcriptional activator, thus upregulating MMP-13 expression (Ju et al., 2004). Thus Hes1 acts to increase expression of MMP-13. It is of particular interest to note that HES1 acts through Notch signaling pathway (Kageyama et al., 2007). Notch has previously been shown to modulate sonic hedgehog signaling and work through primary cilia (Ezratty et al., 2011; Kong et al., 2015). In an apparent unrelated mechanism, Niebler et al. (2015) showed that the transcription factor AP-2e is intimately involved in the upregulation of MMP-13 as OA progresses.

Bardet–Biedl syndrome (BBS, MIM 209900) is a pleiotropic genetically heterogeneous disorder characterized by obesity, retinopathy, polydactyly, renal anomalies including polycystic kidney disease, intellectual disabilities and hypo-genitalism (Chiang et al., 2004; Kaushik et al., 2009; Marion et al., 2011). To date, 21 BBS genes have been identified (Katsanis et al., 2000; Slavotinek and Biesecker, 2000; Mykytyn et al., 2001; Nishimura et al., 2001, 2005; Ansley et al., 2003; Badano et al., 2003; Chiang et al., 2004, 2006; Li et al., 2004; Stoetzel et al., 2006a,b, 2007). Although the cellular functions of BBS proteins are not yet fully understood, evidence from a variety of organisms, including BBS mouse models, demonstrate that BBS proteins play a role in cilia assembly and/or function, as well as intracellular vesicle trafficking. BBS mouse models lack spermatozoa flagella (Fan et al., 2004; Badano et al., 2005). Knock down of multiple BBS genes in zebrafish have been shown to interfere with function of nodal cilia, as well as to result in delay of intracellular vesicle transport. *C. elegans* homologs of Bbs1, Bbs2, Bbs7 and Bbs8 have been shown to be expressed in ciliated cells and Bbs8 was found in the ciliary basal body but not in the microtubule-based ciliary axoneme (Ansley et al., 2003). *BBS1, BBS4, BBS5, BBS7,* and *BBS8* have also been reported to localize to the centrosome and/or basal body of widely used mammalian cell lines (Badano et al., 2005). The depletion of *BBS4* through RNA interference has been shown to lead to the disruption of cytoplasmic microtubule arrays, the mis-localization of some pericentriolar proteins including pericentriolar material 1 protein (PCM1), and cell division arrest and apoptosis in cultured cells (Kim et al., 2004). Recent studies indicate that the seven most conserved BBS proteins (BBS1, BBS2, BBS4, BBS5, BBS7, BBS8, and BBS9), along with BBS18 form a stable complex known as the BBSome. The BBSome associates with centriolar satellites in the cytoplasm and transitions to a membrane-associated form at the base of the cilium. Depletion of some components of the BBSome affects ciliogenesis by interfering with membrane trafficking to the primary cilium (Nachury et al., 2007). Studies involving renal cells have shown a predisposition of primary cilia to act as surface receptors (Marion et al., 2011) and function as both mechano- and chemoreceptors for specific environments (Poole et al., 1985, 1997, 2001; Kouri et al., 1996, 1998; Jensen et al., 2004; McGlashan et al., 2006; Kaushik et al., 2009). For a recent reviews of BBS genes and associated diseases see the following papers (Heon et al., 2016; Kaur et al., 2017; Weihbrecht et al., 2017). We chose to work with mice with the Bbs1^*M390R/M390R*^ since it accounts for 25% of all human BBS cases and is directly involved with the BBSome.

Previous work has visualized the presence of primary cilia on chondrocytes, with each chondrocyte containing a single primary cilium (Scherft and Daems, 1967; Hart, 1968; Wilsman, 1978; Meier-Vismara et al., 1979; Wilsman et al., 1980; Kaushik et al., 2009). One study demonstrated cartilage abnormalities associated with defective primary cilia (Kaushik et al., 2009). Studies employing Oak Ridge polycystic disease mouse chondrocytes show that the primary cilia in chondrocytes are not involved in the initial mechanosensation, but are associated with signal processing in response to increased loads (Mykytyn and Sheffield, 2004). We hypothesized that dysfunctional primary cilia result in OA in Bbs1^*M390R/M390R*^ mice as they age through intra-cellular pathways resulting in up-regulation of the previously identified HTRA1-DDR2-MMP13 degradative pathway common to OA.

## Materials and Methods

### Tissue Samples

Knees from wild type (WT) *n* = 44 (*n* = 8 10–14 weeks old; *n* = 24 15–18 weeks old; *n* = 12 21+ weeks old) and Bbs1^*M390R/M390R*^ *n* = 61 (*n* = 18 10–14 weeks old; *n* = 39 15–18 weeks old; *n* = 4 21+ weeks old) mutant mice were obtained from the laboratory of Val Sheffield at the University of Iowa. Knees were harvested and fixed in 4% paraformaldehyde prior to decalcification and embedding according to a previously reported procedure (Spencer et al., 2015). Knees were serially sectioned and sections analyzed histologically after staining with Safranin O and fast green as previously reported (Spencer et al., 2015). Using a light microscope equipped with a digital camera, photographs of each knee joint were taken at 100× and 200× magnifications. The articular cartilage in two representative sections from each stained slide were analyzed using the OARSI scoring system (Glasson et al., 2010) to quantify the pathological state of each joint, with a score of zero representing unaltered articular cartilage and six representing severe OA.

### Immunohistochemical Analysis

Immunohistochemistry (IHC) was performed on slides representing serial sections of mouse knee joints from all animals. Separate slides were stained with antibodies against HTRA1, DDR-2, MMP-13, and TGF-β1. Each slide was deparaffinized and then blocked with 5% bovine serum albumin for 1 h. Primary antibodies against HTRA-1 (ab38611) (Abcam, Cambridge, MA, United States), DDR-2 (SC-8989) (Santa Cruz Biotechnology, Santa Cruz, CA, United States), MMP-13 (AB8120) (Chemicon, Temecula, CA, United States) and TGF-β1 (Abcam- ab92486, Rabbit Polyclonal IgG) were used. All antibodies were diluted 1:200, applied to specimens, and incubated overnight at 4°C. On the second day, samples were rinsed with PBS and then incubated with an avidin/biotin ABC mix (Vectastain elite ABC Kit). Slides were rinsed again with PBS and incubated with a species appropriate biotinylated secondary antibody. After a third rinse, a color reaction was initiated using a peroxidase substrate (Vector Labs, NovaRED). Negative controls were prepared by staining without the addition of primary antibody. Differences in staining intensity were compared qualitatively with WT controls. Blind counting of stained cells was performed using ImageJ (NIH, Bethesda, MD, United States). IHC and histological analysis focused on different parts of the joint to demonstrate the universal nature of the OA in the Bbs1^*M390R/M390R*^ model. Animals were grouped into three general age categories (10–14 weeks, 15–18 weeks, and 21+ weeks) for comparison purposes. The age categories selected indicate young, middle age, and old mice. For the purposes of IHC and histological analysis, i.e., cell counting (see section “ImageJ Analysis” below), the number of mice used in each category was 10–14 weeks: WT *n* = 8, Bbs1^*M390R/M390R*^ mutant *n* = 18, 15–18 weeks: WT *n* = 24, Bbs1^*M390R/M390R*^ mutant *n* = 39, 21+ weeks: WT *n* = 12, Bbs1^*M390R/M390R*^ mutant *n* = 4.

### Zebrafish Immunohistochemistry

Zebrafish IHC techniques were employed as previously described (Suli et al., 2014). Antibodies used for visualization: Primary cilia: anti-mouse acetylated tubulin (1:500, Sigma, T7451) with anti-mouse IgG Alexa488 (1:400, Invitrogen, A-28175); Primary DDR2: anti-mouse DDR2 (1:200, Santa Cruz SC-8989) with anti-rabbit IgG Alexa405 (1:400, Invitrogen, A31556).

Larvae were fixed with 4% paraformaldehyde either for 2 h at room temperature, washed three times, 20 min each, with PBST (0.1% Tween in PBS) and incubated 1 h in distilled water. They were placed in block solution [1% bovine serum albumin, 1% dimethyl sulfoxide (DMSO), and 0.02% sodium azide in PBST, 10% normal goat serum] for 1 h and then incubated with primary antibody overnight at 4°C. After four 20 min washes with PBST, they were incubated with secondary antibody for 3 h at room temperature, washed four times, 10 min each, in PBST and cleared in 50% glycerol/PBS. Embryos were imaged using an Olympus FV1000 confocal microscope.

### Osteoarthritis Scoring

The articular cartilage in two representative sections from each stained slide was analyzed using the OARSI scoring system to quantify the pathological state of each joint, with a score of zero representing unaltered articular cartilage and six representing severe OA. Overall OARSI scoring was based on an osteoarthritic damage 0–6 subjective scoring system applied to all four quadrants of the knee (Glasson et al., 2010). The articular cartilage in two representative sections from each stained slide was also analyzed using a modified Mankin score to quantify the pathological state of each joint, with a score of zero representing unaltered articular cartilage and 14 representing severe OA. Overall Mankin scoring was based on a subset of scores including cartilage erosion score (0–6), chondrocyte periphery staining (0–2), spatial arrangement of chondrocytes (0–3), and background staining intensity (0–3) (Mankin et al., 1971; Xu et al., 2003; Bomsta et al., 2006). Statistical significance of the combined Mankin scores for the 28-day Bbs1^*M390R/M390R*^ mutant and wild-type surgery groups using a two-way ANOVA test conducted by the Department of Statistics at Brigham Young University. As in the case of IHC, animals were grouped into three general age categories (10–14 weeks, 15–18 weeks, and 21+ weeks) for comparison purposes. The age categories selected indicate young, middle age, and old mice. For the purposes of OARSI scoring analysis the number of mice used in each category was 10–14 weeks: WT *n* = 8, Bbs1^*M390R/M390R*^ mutant *n* = 18, 15–18 weeks: WT *n* = 24, Bbs1^*M390R/M390R*^ mutant *n* = 39, 21+ weeks: WT *n* = 12, Bbs1^*M390R/M390R*^ mutant *n* = 4.

#### ImageJ Analysis

The expression levels of HTRA1, DDR-2, MMP-13, and TGF-β1 were analyzed quantitatively by calculating the percentage of cells staining positive for the respective biomarkers and the total number of chondrocytes in a defined 200×900 pixel area of articular cartilage immediately distal to the tibial plateau (Larkin et al., 2013). All quantitative analysis was performed using ImageJ (National Institutes of Health, Bethesda, MD, United States). Cell counting was done by two independent investigators who were blinded to the strain of mouse (Bbs1^*M390R/M390R*^ mutant vs. WT). The quantitative results were subsequently analyzed statistically using an ANOVA test to detect differences in the mean percentages of positive staining for key OA biomarkers and mean chondrocyte counts between the Bbs1^*M390R/M390R*^ mutant and WT samples. The numbers of animals analyzed for each age group were previously indicated in the IHC methods section.

#### Adobe Photoshop Analysis

To compare the thickness of cartilage between Bbs1^*M390R/M390R*^ mutant vs. WT, cartilage measurements above the subchondral bone for each mouse specimen was captured using the Lasso Tool in *Adobe Photoshop*. This was done to measure the area between the apical surface of the cartilage and where the articular cartilage interfaces with subchondral bone. The technique was perfected in our laboratory and previously reported (Black et al., 2015). To help standardize the human measurement error, two different lab members independently measured the tissue samples.

### Statistics

Statistical analysis was performed using a mixed models analysis of variance (ANOVA) on the OARSI or Mankin score as well as. The dependent variables were the OARSI, Mankin scores or biomarker staining for the knee. The independent variables were age and genotype along with their interaction. Blocking was done on each animal to account for their multiple measures. *Post hoc* *t*-tests were performed to determine differences in genotype biomarker expression level at each age, *p*-values of <0.05 were considered significant. The percent of chondrocyte biomarker expression level staining was compared using *t*-tests. A Bonferroni adjust was made for these four tests. Thus *p*-values of <0.0125 were considered significant.

## Results

### Co-expression of DDR-2 and Primary Cilia

**FIGURE 1:**
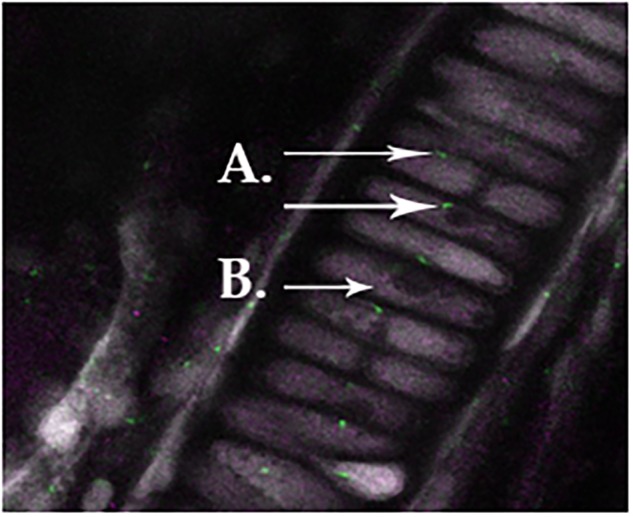
Normal primary cilia in zebrafish chondrocytes residing in the mandible, as indicated in green staining for acetylated α-tubulin **(A)**. Ddr-2 is indicated by purple staining **(B)**, demonstrating a lack of co-localization between primary cilia and Ddr-2.

We observed primary cilia (acetylated α-tubulin) in zebrafish chondrocytes residing in the mandible (**Figure 1A**) indicated in green staining. DDR-2, indicated by purple staining (**Figure 1B**), did not co-localize with primary cilia.

### Cartilage Morphology

**FIGURE 2:**
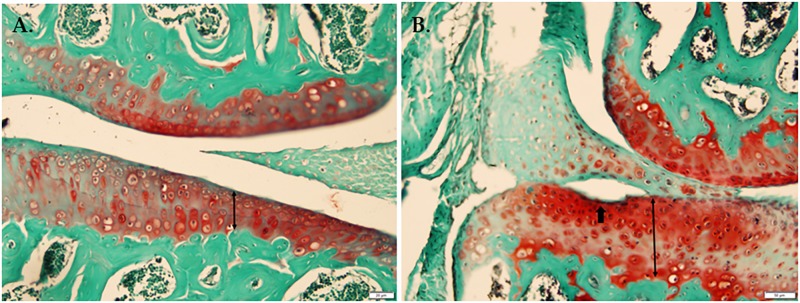
Histological comparison of WT and Bbs1^*M390R/M390R*^ mutant knee joints. **(A)** Knee joint stained with Safranin-O Fast Green from a 17-week-old male WT mouse. **(B)** Knee joint stained with Safranin-O, Fast Green from an 18-week-old male Bbs1^*M390R/M390R*^ mutant mouse. Note the surface erosion and exaggerated thickness of cartilage. Bbs1^*M390R/M390R*^ mutant knees exhibited significantly thicker cartilage compared to WT controls (*p* < 0.01). However, Bbs1^*M390R/M390R*^ mutant mice do not exhibit higher than normal OARSI or Mankin scores.

In a histological comparison of 18-week-old WT and Bbs1^*M390R/M390R*^ mutant knee joints stained with Safranin-O, Fast Green we observed thicker cartilage (*p* < 0.01) in Bbs1^*M390R/M390R*^ mutant knees with noted surface erosion in some cases. See **Figure 2** for representative pictures.

### OARSI and Mankin Scores

**FIGURE 3:**
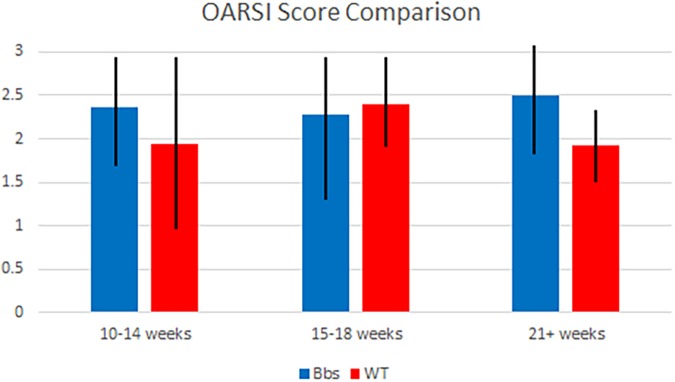
OARSI score comparison of Bbs1^*M390R/M390R*^ mutant and WT mice at various ages. Articular cartilage degradation from both mutant and WT samples was objectively evaluated using the OARSI scoring system by two blind scorers. Samples were combined into generalized age groups of 10–14 (WT *n* = 8; Bbs1^*M390R/M390R*^ mutant *n* = 18), 15–18 (WT *n* = 24; Bbs1^*M390R/M390R*^ mutant *n* = 39) and 21+ (WT *n* = 12; Bbs1^*M390R/M390R*^ mutant *n* = 4) weeks. Mean OARSI scores of 2.25, 2.2, and 2.5 (10–14, 15–18, 21+ weeks, respectively) were not significantly different than those of WT mice.

Although Bbs1^*M390R/M390R*^ mutants tended to have higher OARSI scores than WT mice (**Figure 3**), the mixed models analysis showed no statistically significant difference was observed in OARSI or Mankin (data not shown) scoring between the two groups studied. The alteration from normal biomarker concentrations in Bbs1^*M390R/M390R*^ mutant mice, suggests that the metabolic changes occur prior to cartilage degradation.

### MmMP-13, HTRA1, and TGF-β1 Expression in *Bbs1*^*M390R/M390R*^ Mutant Mice

**FIGURE 4:**
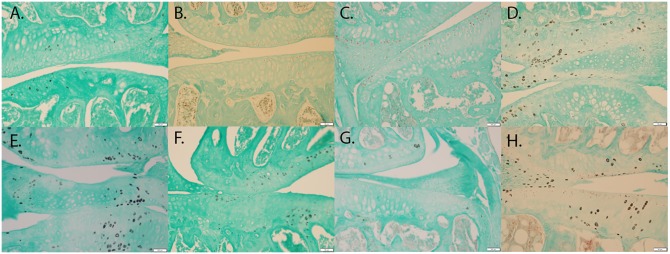
Representative images showing the results of the immunohistochemical and histological staining performed to analyze the presence of OA biomarkers. Although, Bbs1^*M390R/M390R*^ mutant and WT mouse knees did not exhibit a statistical difference in cartilage erosion scoring, we analyzed separately cell staining within the three major age (week) categories examined – 10–14 (WT *n* = 8; Bbs1^*M390R/M390R*^ mutant *n* = 18), 15–18 (WT *n* = 24; Bbs1^*M390R/M390R*^ mutant *n* = 39), 21+ (WT *n* = 12; Bbs1^*M390R/M390R*^ mutant *n* = 4). All tissue samples represented in this figure were age matched at 14 weeks. The patterns of cell staining for biomarkers examined did not differ significantly with age. **(A)** WT MMP-13; **(B)** WT HTRA-1; **(C)** WT TGF-β1; **(D)** WT DDR-2; **(E)** Bbs1^*M390R/M390R*^ mutant MMP13; **(F)** Bbs1^*M390R/M390R*^ mutant HTRA-1; **(G)** Bbs1^*M390R/M390R*^ mutant TGF-β1; **(H)** Bbs1^*M390R/M390R*^ mutant DDR-2.

*T*-tests of immunohistochemical staining revealed significant differences in the percent of chondrocytes staining positive for MMP-13 (*p* < 0.001) (**Figure 4A** vs. **Figure 4E**), HTRA1 (*p* < 0.001) (**Figure 4B** vs. **Figure 4F**), and TGF-β1 (*p* < 0.001) (**Figure 4C** vs. **Figure 4G**), but not DDR-2 (*p* > 0.05) (**Figure 4D** vs. **Figure 4H**) was not significant with the Bonferroni adjustment, in Bbs1^*M390R/M390R*^ mutant mice when compared to age matched WT mice. These findings remained consistent across all ages examined.

When comparing all age groups, WT mice exhibited significantly less staining for HTRA1 and MMP-13 compared to Bbs1^*M390R/M390R*^ mutants. WT mice exhibited significantly more staining for TGF-β1 compared to Bbs1^*M390R/M390R*^ mutants, indicative of earlier stages of OA. This is consistent with previous observations of an inverse relationship between HRTA1 and TGF-β1 (Larkin et al., 2013). We did not observe a decrease in DDR2 expression in Bbs1^*M390R/M390R*^ mutant mice compared to WT controls (**Figure 4D** vs. **Figure 4H**), contrary to general observations of upregulation in OA cartilage compared to controls. Notwithstanding the verified catabolic role of DDR2, no protection was afforded Bbs1^*M390R/M390R*^ mutant cartilage by its decreased expression. Visualization of the primary cilia (acetylated α-tubulin) and DDR2 in embryonic mandibular zebrafish chondrocytes (**Figure 1A**) indicated that DDR2 did not co-localize with primary cilia. The data are summarized in **Table 1**.

**Table 1:** Percent Bbs1^*M390R/M390R*^ mutant cells stained with biomarkers compared with WT.

	Bbs1^*M390R/M390R*^	WT
**HTRA-1**		
Week 10–14	86%^∗^	39%
Week 15–18	100%^∗^	50%
Week 21+	100%^∗^	61%
**MMP-13**		
Week 10–14	93%^∗^	13%
Week 15–18	100%^∗^	14%
Week 21+	100%^∗^	67%
**TGF-βl**		
Week 10–14	50%^∗^	79%
Week 15–18	80%^∗∗^	100%
Week 21+	98%	100%

^∗^*P* < 0.001; ^∗∗^*P* < 0.05.

## Discussion

It is imperative to fully understand the metabolic pathway involved in the age-related onset and progression of OA if an effective treatment is to be developed. Common to the pathogenesis of human OA is the activation of the HTRA1-DDR2-MMP13 axis. HTRA1 is strongly expressed in the presence of stressors in murine OA models, and its activation augments the expression of DDR-2 (Polur et al., 2010). Upregulation of DDR2 results in excess binding of the receptor to its ligand, type II collagen (Leitinger et al., 2004), which in turn stimulates high levels of expression of the MMP-13 (Su et al., 2009). This ultimately leads to the destruction of articular cartilage. Aside from the wide array of evidence showing HTRA1-DDR2-MMP13 activity in OA of multiple modalities, the convergence of diverse noxious stimuli upon this axis is further supported in the protective effects exerted in DDR-2 hypomorphic strains of mice. When subjected to the destabilization of the medial meniscus (DMM) surgical procedure they demonstrate a significant decrease in the progression of OA compared to WT littermates (Xu et al., 2010). We aimed to demonstrate the significance of primary cilia as it pertains to the onset and progression of OA. It is noteworthy that ciliopathy related genes do not just function in cilia. For example, the BBSome plays a role in leptin receptor trafficking and insulin receptor trafficking to the plasma membrane in neurons in the hypothalamus. Thus, BBS genes may play a role in OA pathways even if not directly involving the primary cilia organelle. Curiously, WT mice consistently exhibited significantly more staining for DDR-2 than Bbs1^*M390R/M390R*^ mutants at all age observed age groups, inconsistent with previous observations correlating high levels of DDR-2 expression with more advanced OA. This may suggest a role for primary cilia in processing signals associated with DDR-2 activation and/or expression of the receptor.

In order to identify primary cilia as a component in the chondrocyte destruction pathway associated with OA, we used Bbs1^*M390R/M390R*^ mutant mice, which are known to have defective primary cilia (**Figure 2B**) (Mykytyn et al., 2004) and have been previously shown to exhibit OA-like phenotype (Kaushik et al., 2009). We compared Bbs1^*M390R/M390R*^ mutants to WT at different ages and in each instance found significant differences in the concentrations of key biomarkers previously shown to be associated with OA. Among these differences were increased concentrations of chondrocytes expressing HTRA-1 and a decreased concentration of chondrocytes expressing TGF-b1 and increased levels of MMP-13. All three observations are consistent with previous studies on biomarkers associated with OA (Murwantoko et al., 2004; Polur et al., 2010; Holt et al., 2012; Larkin et al., 2013). The increased presence of HTRA1 and MMP13 may help explain why the Bbs1^*M390R/M390R*^ mutants respond more vigorously to DMM surgery (Peterson et al., 2011).

TGFβ-1 functions largely in the SMAD signaling pathway; it is a cytokine involved in a number of cellular processes including cell growth, proliferation, differentiation, apoptosis, and the elaboration of extracellular matrix (van Beuningen, 2000). HTRA-1 has a degrading effect on the peri- and extracellular matrix, as well as an inhibitory effect on TGF-β1 (Oka et al., 2004; Tocharus et al., 2004). Thus HTRA-1 may contribute to OA not only through direct matrix destruction, but also by inhibiting possible protection to the matrix offered by TGF-β1. Expression of MMP-13, a protein with a degrading effect on extracellular matrix similar to HTRA-1, appears to be upregulated. This is consistent with alterations in MMP-13 expression as in OA cases resulting from other causes, such as aging, obesity, joint misalignment, and acute injury.

Our results indicate that age-related OA-associated expression of DDR-2 is altered in Bbs1^*M390R/M390R*^ mice. Curiously, DDR-2 expression appeared to be lower in Bbs1^*M390R/M390R*^ mutant mice than in WT mice at all ages. Whereas in cases of OA resulting from the aforementioned etiologies, DDR-2 expression is upregulated in mice presenting with OA. We believe a possible explanation for this could be that primary cilia might have a role in processing signals associated with DDR-2 activation and/or expression of the receptor. However, we have demonstrated that DDR2 does not co-localize to primary cilia (**Figure 2A**). Therefore, any interaction(s) between primary cilia and DDR2 would presumably be indirect.

We did not observe a statistical difference between Bbs1^*M390R/M390R*^ mutant and WT mice when using knee histological scoring methods (OARSI and Modified Mankin). We did observe a difference in the thickness of cartilage in Bbs1^*M390R/M390R*^ mutant mice compared to WT as others have previously noted (Kaushik et al., 2009).

Finally, much has been published regarding the potential role of inflammation in OA. More recently, the interaction of adipokines and inflammation in initiating OA has been demonstrated (Azamar-Llamas et al., 2017). Leptin, a peptide hormone involved in maintaining insulin sensitivity and contributing to the sensation of satiety, is expressed at very high levels in obese individuals. It appears to be correlated with OA as well, with intervention at the level of MMP-13 expression occurring. Downregulation of leptin mRNA translation via small interference RNA molecules inhibits MMP-13 expression in cultured osteoarthritic chondrocytes (Iliopoulos et al., 2007; Pallu et al., 2010). The situation appears to be most exacerbated in cases of extreme obesity where a strong positive correlation exists between the responsiveness of the *MMP-13* gene to leptin and the BMI of osteoarthritic individuals. This is intriguing in light of the observation that primary cilia are intimately involved in leptin homeostasis (Seo et al., 2009; Guo et al., 2016).

## Conclusion

Our findings support our initial hypothesis that BBS mutations are involved in the identified molecular pathway common to OA, with the exception of DDR-2. Since the BBSome plays a role in leptin receptor trafficking and insulin receptor trafficking to the plasma membrane in neurons in the hypothalamus, BBS genes may play a role in OA pathways even if not directly involving the primary cilia organelle. We have shown that MMP-13, HTRA-1, and TGF-β1 expression mimic their expression during all other investigated cases of OA. This suggests that primary cilia act as a surface receptor on chondrocytes and work to relay the condition of the cartilaginous matrix and its integrity to the associated chondrocyte. Primary cilia may also be an important link between inflammatory signals and OA. Future research should work to identify the specific role primary cilia play in the onset and progression of OA.

## Strengths And Limitations

This paper adds comprehensive and important data to a paucity of information regarding the involvement of primary cilia in the pathogenesis of OA. Some significant and important findings in this work are that primary cilia are a link between pro-inflammatory bio-markers and OA and that the BBSome may play a role in this disease as well. While BBS is a pleiotropic disease with 21 known genes, the work associated with this paper employed the BBS mutant (Bbs1^*M390R/M390R*^) which is associated with 25% of all human cases. Some weaknesses of this work include the lack of elucidating the exact molecular pathway(s) or link(s) between primary cilia and OA as well as no data from human BBS patients.

## Institutional Safety

All U.S. National Institutes of Health guidelines for research involving Recombinant or Synthetic Nucleic Acid Molecules were followed. All chemical use was according to the U.S. Occupational Safety and Health Administration guidelines as overseen by the Brigham Young University Office of Risk Management.

## Ethics Statement

This study was performed in strict accordance with the recommendations in the Guide for the Care and Use of Laboratory Animals of the National Institutes of Health. All of the animals were handled according to approved Institutional Animal Care and Use Committee (IACUC) protocols 5061426 and 6081818, of the University of Iowa and the Brigham Young University Institutional Animal Care and Use Committee protocol 13-0601. Animals were housed according to IACUC recommendations. Methods of euthanasia used were carbon dioxide inhalation followed by cervical dislocation, or anesthesia induced by ketamine/xylazine followed by transcardial perfusion. Humane endpoints were strictly observed, and every effort was made to minimize suffering.

## Author Contributions

IS provided direct oversight for the project. MM, SG, DB, JC, BD, CW, BR, AS, MG, and JD performed all of the histological work including analyses associated therewith. DE was responsible for all statistical work assisted by MG. DE also assisted with experimental design. VS provided the BBS mice and assisted with experimental design. AS provided the zebrafish and oversaw all of that work, assisted by SG and JD. DK conceived the project and was the overall coordinator for it.

## Conflict of Interest Statement

The authors declare that the research was conducted in the absence of any commercial or financial relationships that could be construed as a potential conflict of interest.
